# Urinary N-terminal pro–B-type natriuretic peptide as a biomarker for cardiovascular events in a general Japanese population: the Hisayama Study

**DOI:** 10.1186/s12199-021-00970-0

**Published:** 2021-04-12

**Authors:** Keisuke Yamasaki, Jun Hata, Tomomi Ide, Takuya Nagata, Satoko Sakata, Daigo Yoshida, Takanori Honda, Yoichiro Hirakawa, Toshiaki Nakano, Takanari Kitazono, Hiroyuki Tsutsui, Toshiharu Ninomiya

**Affiliations:** 1 Department of Epidemiology and Public Health, Graduate School of Medical Sciences, Kyushu University, Fukuoka, Japan; 2 Department of Medicine and Clinical Science, Graduate School of Medical Sciences, Kyushu University, Fukuoka, Japan; 3 Center for Cohort Studies, Graduate School of Medical Sciences, Kyushu University, Fukuoka, Japan; 4 Department of Cardiovascular Medicine, Graduate School of Medical Sciences, Kyushu University, Fukuoka, Japan

**Keywords:** Biomarker, Cardiovascular disease, General populations, Prospective study, Urinary NT-proBNP

## Abstract

**Background:**

Epidemiological evidence has shown that serum N-terminal pro-brain natriuretic peptide (NT-proBNP) concentrations, a diagnostic biomarker for heart failure, are positively associated with cardiovascular risk. Since NT-proBNP in serum is excreted in urine, it is hypothesized that urinary NT-proBNP concentrations are correlated with serum concentrations and linked with cardiovascular risk in the general population.

**Methods:**

A total of 3060 community-dwelling residents aged ≥ 40 years without history of cardiovascular disease (CVD) were followed up for a median of 8.3 years (2007–2015). Serum and urinary concentrations of NT-proBNP at baseline were compared. The hazard ratios (HRs) and their 95% confidence intervals (CIs) for the association between NT-proBNP concentrations and the risk of developing CVD were computed using the Cox proportional hazards model.

**Results:**

The median values (interquartile ranges) of serum and urinary NT-proBNP concentrations at baseline were 56 (32–104) pg/mL and 20 (18–25) pg/mL, respectively. There was a strong quadratic correlation between the serum and urinary concentrations of NT-proBNP (coefficient of determination [*R*^2^] = 0.72): urinary concentrations of 20, 27, and 43 pg/mL were equivalent to serum concentrations of 55, 125, and 300 pg/mL, respectively. During the follow-up period, 170 subjects developed CVD. The age- and sex-adjusted risk of CVD increased significantly with higher urinary NT-proBNP levels (*P* for trend < 0.001). This association remained significant after adjustment for traditional cardiovascular risk factors (*P* for trend = 0.009). The multivariable-adjusted risk of developing CVD almost doubled in subjects with urinary NT-proBNP of ≥ 43 pg/mL as compared to those with urinary NT-proBNP of ≤ 19 pg/mL (HR 2.07, 95% CI 1.20–3.56).

**Conclusions:**

The present study demonstrated that urinary NT-proBNP concentrations were well-correlated with serum concentrations and were positively associated with cardiovascular risk. Given that urine sampling is noninvasive and does not require specially trained personnel, urinary NT-proBNP concentrations have the potential to be an easy and useful biomarker for detecting people at higher cardiovascular risk.

**Supplementary Information:**

The online version contains supplementary material available at 10.1186/s12199-021-00970-0.

## Introduction

N-terminal pro–B-type natriuretic peptide (NT-proBNP) is a stable N-terminal fragment of proBNP that is secreted from ventricular myocytes in response to mechanical ventricular wall stretch and ischemic injury [1]. Serum NT-proBNP has been well accepted as an established biomarker for the diagnosis and prognosis of patients with heart failure [2–4]. In addition, several population-based studies have reported that higher serum NT-proBNP levels are significantly associated with greater risk of cardiovascular disease (CVD), suggesting that serum NT-proBNP has potential as a biomarker for estimating future cardiovascular risk in general populations [5–8].

Since NT-proBNP is known to be filtered in the glomeruli and excreted in urine [9–11], it seems reasonable to expect that urinary NT-proBNP concentrations would reflect the serum concentration of NT-proBNP secreted from the myocardium [12]. In support of this idea, several hospital-based studies have reported that the urinary concentration of NT-proBNP showed a strong correlation with serum/plasma NT-proBNP levels among patients with heart failure or hypertension [13–21]. Since urine sampling is less invasive and easier than serum sampling, urinary NT-proBNP concentrations would have potential as a biomarker for self-monitoring of the status of cardiovascular risk and/or heart failure [22], if tools could be developed for the measurement of urinary NT-proBNP, similar to the self-monitoring devices for the urinary sodium-to-potassium ratio [23]. However, it remains unclear whether serum and urinary concentrations of NT-proBNP are correlated in general populations, which have relatively lower serum NT-proBNP concentrations than patients with heart failure. Moreover, to our knowledge, there have been no studies investigating the association of urinary NT-proBNP concentrations with cardiovascular risk in general populations.

The aims of the present study were to evaluate the correlation between serum and urinary concentrations of NT-proBNP and to investigate the association between urinary NT-proBNP levels and the risk of CVD in a general Japanese population.

## Methods

### Study population

A population-based prospective cohort study of CVD and its risk factors have been established since 1961 in the town of Hisayama, a suburb of the Fukuoka metropolitan area of Kyushu Island in Japan. The population of Hisayama was approximately 8400 in 2010, and full community surveys of the health status of residents ≥ 40 years of age have been repeated since 1961. Details of this study have been described elsewhere [24, 25]. In brief, a total of 3384 residents aged ≥ 40 years participated in the health examination in 2007–2008 (participation rate: 78.2%). We excluded individuals who did not consent to participate in the study (*n* = 8), those with a history of CVD (*n* = 223), and those without urinary or serum samples (*n* = 93), and thus, the remaining 3060 participants were enrolled in the present study.

### Follow-up survey

The participants of the Hisayama Study were followed up prospectively for a median of 8.3 years from the date of comprehensive assessment in 2007–2008 to November 2015 by repeated annual health examinations. Their health status was checked yearly by mail or telephone for participants who did not undergo a regular examination or who had moved away from town. In addition, a daily monitoring system was established among the study team consisting of local physicians and members of the town’s Health Office. When a new onset of CVD including stroke and coronary heart disease (CHD) occurred or was suspected in the follow-up survey, we collected all the relevant medical information including hospital charts, physicians’ records, physical and neurological examinations, laboratory examinations, and death certificate, and then reviewed them to determine the onset of CVD. In addition, when a participant died, an autopsy was performed at the Department of Pathology of Kyushu University if consent for autopsy was obtained. During the follow-up period, all participants were followed up completely, and 318 participants died, of whom 185 (58.2%) underwent autopsy examinations.

### Measurements of serum and urinary NT-proBNP

At the screening examination, blood samples were collected from an antecubital vein. Urine samples were also collected at the time of the health checkup visit. A portion of each serum and urinary sample was stored at – 80 °C until the measurement of NT-proBNP concentrations in 2009 and 2018, respectively. Serum and urinary concentrations of NT-proBNP were determined using the Elecsys proBNP Immunoassay (Elecsys 2010 from Roche Diagnostics, Switzerland) [26]. The assay limit of detection was 5 pg/mL. For 20 subjects whose serum NT-proBNP concentrations were lower than the assay limit, the serum concentrations were defined as 5 pg/mL for statistical analyses. No subjects had urinary NT-proBNP concentrations lower than the assay limit.

### Outcomes

The main outcome of the present study was the development of total CVD defined as a first-ever episode of either stroke or CHD. The alternative outcomes were the subtypes of CVD—namely, CHD and stroke. CHD represented acute or silent myocardial infarction, percutaneous transluminal coronary angioplasty, coronary artery bypass graft surgery, or sudden cardiac death within 1 h. Stroke was defined as a sudden onset of nonconvulsive and focal neurological deficit persisting for > 24 h. The diagnostic criteria for the outcomes were described in detail previously [24, 25].

### Other risk factor measurements

At the baseline examination, each participant answered a self-administered questionnaire concerning the current use of antihypertensive agents, insulin, oral glucose-lowering agents, and lipid-lowering medication; smoking habits; alcohol intake; and physical activity, and the questionnaire was checked by trained interviewers. Smoking habits and alcohol intake were categorized as current use or no current use. Regular exercise was defined as participation in sports three or more times a week during leisure time. Blood pressure was measured three times using an automated sphygmomanometer after the participants had rested for at least 5 min in the sitting position. The mean of the three measurements was applied for the present analysis. Hypertension was defined as systolic blood pressure levels ≥ 140 mmHg, diastolic blood pressure ≥ 90 mmHg, and/or current treatment with antihypertension agents. Plasma glucose levels were measured by the hexokinase method. Diabetes mellitus was determined by a fasting plasma glucose level of ≥ 7.0 mmol/L, 2-h 75-g oral glucose postloaded or casual glucose levels of ≥ 11.1 mmol/L, and/or current use of oral glucose-lowering agents or insulin. Serum total cholesterol and high-density lipoprotein cholesterol levels were measured enzymatically. Hypercholesterolemia was defined as serum total cholesterol levels of ≥ 5.69 mmol/L or current use of lipid-lowering medication. Serum creatinine concentrations were measured using the enzymatic method. The estimated glomerular filtration rate (eGFR) was calculated using the Chronic Kidney Disease Epidemiology (CKD-EPI) Collaboration equation with a Japanese coefficient of 0.813 [27]. Body height and weight were measured in light clothing without shoes. Body mass index (BMI) was calculated as weight in kilograms divided by height in meters squared. Electrocardiogram abnormalities were defined as left ventricular hypertrophy (Minnesota Code 3-1), ST depression (4-1, 2, 3), or atrial fibrillation (8-3).

### Statistical analysis

To evaluate the correlation between the serum and urinary NT-proBNP concentrations, the following three models were adopted: (model 1) a linear regression between the serum and urinary NT-proBNP levels; (model 2) a linear regression between the log-transformed serum and urinary NT-proBNP levels; and (model 3) a quadratic regression between the log-transformed serum and urinary NT-proBNP levels (Supplementary Table 1). Since the serum and urinary NT-proBNP concentrations exhibited skewed distributions, the log-transformed (log_10_) values were used in the latter two models. The strength of correlation for each model was evaluated by the coefficient of determination (i.e., the square of the Pearson’s correlation coefficient [*R*^2^]). Since the correlation was the strongest in the model 3 (*R*^2^ = 0.72), the cutoff values for urinary NT-proBNP values were determined on the basis of the quadratic equation developed in this model.

Urinary NT-proBNP levels were classified into 4 categories based on the cutoff values of 20, 27, and 43 pg/mL, which were equivalent to serum NT-proBNP concentrations of 55, 125, and 300 pg/mL [2, 3, 5] on the basis of the abovementioned quadratic equation (model 3). The linear trends in the age- and sex-adjusted mean values and frequencies of risk factors across the urinary NT-proBNP levels were tested by the linear and logistic regression analysis, respectively. The age- and sex-adjusted cumulative incidence of CVD across the urinary NT-proBNP levels and its linear trend were tested using the Cox proportional hazards model. The hazard ratios (HRs) with their 95% confidence intervals (CIs) were also estimated using the Cox proportional hazards model. The heterogeneity in the association between subgroups was evaluated by adding multiplicative interaction terms to the relevant Cox model. In the multivariable-adjusted analysis, the risk estimates were adjusted for the traditional cardiovascular risk factors—namely, age, sex, systolic blood pressure, use of antihypertensive agents, diabetes mellitus, serum total cholesterol, high-density lipoprotein cholesterol levels, lipid-lowering agents, BMI, electrocardiogram abnormalities, eGFR, smoking habits, alcohol intake, and regular exercise.

Two-sided *P* values < 0.05 were considered to be statistically significant in all analyses. All statistical analyses were carried out using the SAS statistical software program, version 9.4 (SAS Institute).

## Results

### Correlation between serum and urinary NT-proBNP concentrations

**Fig. 1:**
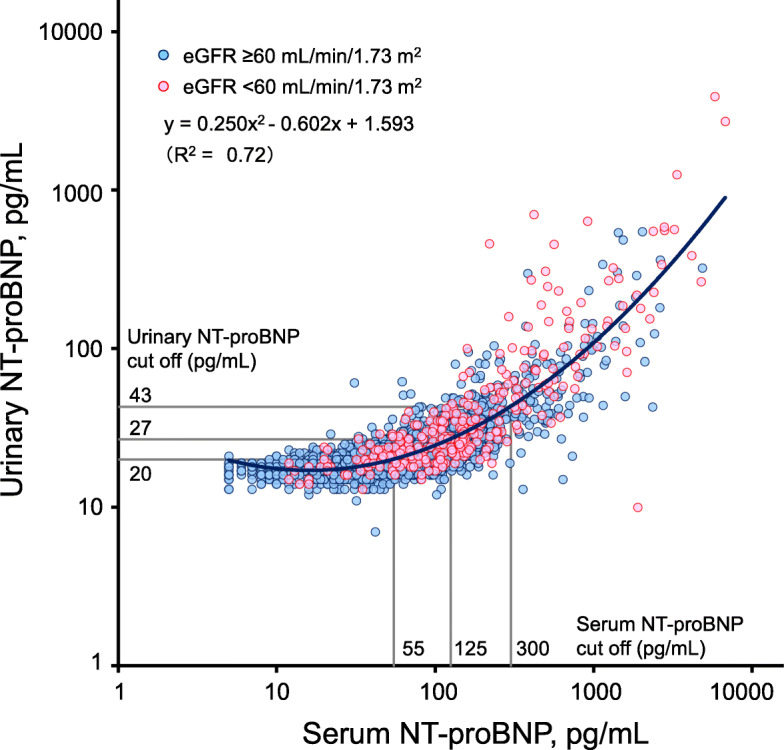
Correlation between serum and urinary concentrations of NT-proBNP. Abbreviations: *NT-proBNP*, N-terminal pro–B-type natriuretic peptide; *eGFR*, estimated glomerular filtration rate. The horizontal axis (serum NT-proBNP) and the vertical axis (urinary NT-proBNP) are log scaled because of their skewed distributions. In the quadratic equation, *x* and *y* indicate the log-transformed (log_10_) concentrations of serum and urinary NT-proBNP, respectively. The quadratic regression curve is shown in blue. Urinary NT-proBNP concentrations corresponding to the guideline-based cutoff values of serum NT-proBNP (55, 125, and 300 pg/mL) were 20, 27, and 43 pg/mL, respectively

The baseline characteristics of population-based samples are shown in Table 1. The median values (interquartile range) of serum and urinary NT-proBNP concentrations in the study participants were 56 (32–104) pg/mL and 20 (18–25) pg/mL, respectively. The scatter plot for the log-transformed concentrations of serum and urinary NT-proBNP in this population is shown in Fig. 1. The urinary concentration of NT-proBNP was positively and strongly correlated with its serum concentration (*R*^2^ = 0.72, *P* < 0.001) and fitted by the following quadratic equation: *y* = 0.250*x*^2^ − 0.602*x* + 1.593, where *x* and *y *are the log-transformed (log_10_) concentrations of serum and urinary NT-proBNP, respectively. Since NT-proBNP is excreted by the kidneys, the correlation between the serum and urinary NT-proBNP concentrations was examined in the subgroups according to eGFR level. Similar distributions and correlations between serum and urinary NT-proBNP concentrations were observed in both subgroups (*R*^2^ = 0.67 for the subgroup with eGFR ≥ 60 mL/min/1.73 m^2^, *R*^2^ = 0.74 for the subgroup with eGFR < 60 mL/min/1.73 m^2^).

**Table 1:** Age- and sex-adjusted clinical characteristics according to the urinary NT-proBNP levels at baseline

Risk factors	Overall (*n* = 3060)	Urinary NT-proBNP levels (pg/mL)				*P* for trend
		≤ 19 (*n* = 1354)	20–26 (*n* = 1105)	27–42 (*n* = 369)	≥ 43 (*n* = 232)	
Age, mean (SE), years	63 (0.2)	57 (0.3)	64 (0.3)	72 (0.6)	78 (0.7)	< 0.001
Women, %	57.9	59.8	58.3	56.3	47.5	0.007
Systolic blood pressure, mean (SE), mmHg	132 (0.3)	132 (0.5)	131 (0.6)	131 (1.0)	133 (1.3)	0.77
Diastolic blood pressure, mean (SE), mmHg	79 (0.2)	80 (0.3)	79 (0.3)	79 (0.6)	78 (0.8)	0.01
Antihypertensive agents, %	29.3	24.8	28.0	27.0	30.6	0.10
Hypertension, %	47.3	45.7	46.4	49.8	54.1	0.06
Diabetes mellitus, %	15.0	14.5	14.2	12.8	13.9	0.61
Serum total cholesterol, mean (SE), mmol/L	5.43 (0.02)	5.53 (0.03)	5.43 (0.03)	5.29 (0.05)	5.08 (0.06)	< 0.001
Serum HDL cholesterol, mean (SE), mmol/L	1.74 (0.01)	1.70 (0.01)	1.76 (0.01)	1.80 (0.02)	1.75 (0.03)	0.004
Lipid-lowering agents, %	13.9	12.3	12.7	9.0	8.8	0.04
Hypercholesterolemia, %	47.3	51.8	47.5	39.2	30.8	< 0.001
Body mass index, mean (SE), kg/m^2^	23.0 (0.1)	23.3 (0.1)	23.1 (0.1)	22.5 (0.2)	21.8 (0.2)	< 0.001
Body mass index ≥ 25 kg/m^2^, %	25.8	27.4	26.5	21.0	19.4	0.006
Electrocardiogram abnormality, %	15.2	11.0	11.6	18.5	36.6	< 0.001
eGFR, mean (SE), mL/min/1.73 m^2^	75 (0.2)	75 (0.2)	75 (0.3)	74 (0.5)	70 (0.6)	< 0.001
eGFR < 60 mL/min/1.73 m^2^, %	10.2	4.2	4.4	7.4	11.0	< 0.001
Current smoking, %	20.1	12.7	14.6	19.3	16.4	0.01
Current drinking, %	48.1	48.7	46.9	45.6	50.6	0.79
Regular exercise, %	12.0	12.1	12.6	11.7	6.8	0.10
Serum NT-proBNP, median (IQR), pg/mL^a^	56 (32–104)	33 (21–52)	65 (44–97)	135 (98–187)	424 (237.5–883.5)	-
Urinary NT-proBNP, median (IQR), pg/mL^a^	20 (18–25)	17 (16–18)	22 (21–24)	31 (28–35)	70.5 (50.5–117.5)	-

*Abbreviations*: *NT-proBNP* N-terminal pro–B-type natriuretic peptide, *SE* Standard error, *HDL* High-density lipoprotein, *eGFR* Estimated glomerular filtration rate, *IQR* Interquartile range

^a^Values were not adjusted for age and sex

### Association of urinary NT-proBNP levels with cardiovascular risk

The age- and sex-adjusted baseline characteristics of the participants according to the urinary NT-proBNP levels are summarized in Table 1. The mean values of age and high-density lipoprotein cholesterol and the frequencies of electrocardiogram abnormalities, subjects with eGFR < 60 mL/min/1.73 m^2^, and current smokers increased significantly with higher urinary NT-proBNP levels. On the other hand, the mean values of diastolic blood pressure, serum total cholesterol, BMI, and eGFR and the frequencies of women, hypercholesterolemia, use of lipid-lowering agents, and subjects with BMI of ≥ 25 kg/m^2^ decreased significantly with higher urinary NT-proBNP levels.

**Fig. 2:**
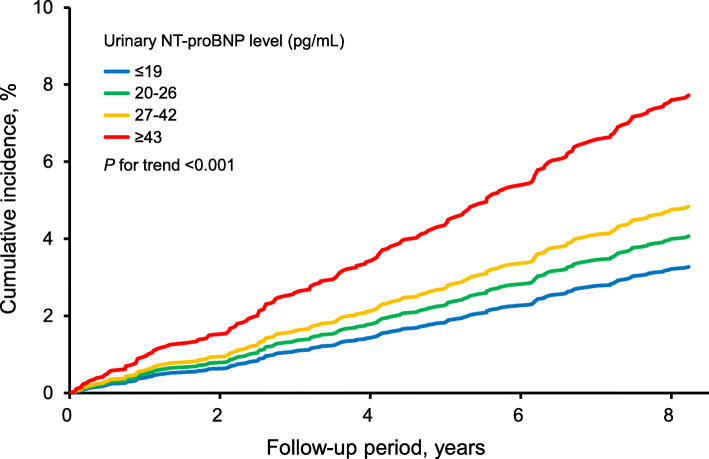
Age- and sex-adjusted cumulative incidence of total cardiovascular disease according to the urinary NT-proBNP levels. Abbreviation: *NT-proBNP*, N-terminal pro–B-type natriuretic peptide

During a median 8.3 years of follow-up, 170 subjects developed CVD. There were 78 cases of CHD and 94 of stroke. The age- and sex-adjusted cumulative incidence of CVD increased significantly with higher urinary NT-proBNP levels (*P* for trend < 0.001) (Fig. 2). The multivariable-adjusted risk of developing CVD increased significantly with higher urinary NT-proBNP levels (*P* for trend = 0.009) (Table 2). With regard to the subtypes of CVD, increased urinary NT-proBNP levels were significantly associated with higher multivariable-adjusted risks of stroke (*P* for trend = 0.004), but no clear association was observed for CHD (*P* for trend = 0.52).

**Table 2:** Age- and sex-adjusted and multivariable-adjusted hazard ratios for the development of cardiovascular disease and its subtypes according to urinary NT-proBNP levels

Urinary NT-proBNP (pg/mL)	Persons at risk	No. of events	Age- and sex-adjusted		Multivariable-adjusted^a^	
			Hazard ratio (95% CI)	*P* value	Hazard ratio (95% CI)	*P* value
**Cardiovascular disease**						
≤ 19	1354	38	1.00 (reference)		1.00 (reference)	
20–26	1105	59	1.25 (0.82–1.90)	0.30	1.30 (0.86–1.99)	0.22
27–42	369	34	1.49 (0.90–2.47)	0.12	1.51 (0.91–2.51)	0.11
≥ 43	232	39	2.41 (1.44–4.04)	< 0.001	2.07 (1.20–3.56)	0.009
*P* for trend				< 0.001		0.009
**Coronary heart disease**						
≤ 19	1354	23	1.00 (reference)		1.00 (reference)	
20–26	1105	26	0.98 (0.55–1.75)	0.93	1.01 (0.56–1.82)	0.97
27–42	369	18	1.54 (0.77–3.04)	0.22	1.52 (0.76–3.06)	0.24
≥ 43	232	11	1.33 (0.59–3.00)	0.49	1.09 (0.45–2.63)	0.85
*P* for trend				0.28		0.52
**Stroke**						
≤ 19	1354	15	1.00 (reference)		1.00 (reference)	
20–26	1105	34	1.73 (0.93–3.21)	0.09	1.76 (0.95–3.29)	0.07
27–42	369	17	1.67 (0.79–3.51)	0.18	1.66 (0.79–3.51)	0.18
≥ 43	232	28	3.81 (1.86–7.80)	< 0.001	3.24 (1.54–6.85)	0.002
*P* for trend				< 0.001		0.004

*Abbreviations*: *NT-proBNP* N-terminal pro–B-type natriuretic peptide, *CI* Confidence interval

^a^Adjusted for age, sex, systolic blood pressure, antihypertensive agents, diabetes mellitus, serum total and high-density lipoprotein cholesterol levels, lipid-lowering agents, body mass index, electrocardiogram abnormality, estimated glomerular filtration rate, smoking habits, alcohol intake, and regular exercise. Five participants with missing data for covariates were excluded from the multivariable-adjusted analysis (*n* = 3055)

Every 1-SD increment in log-transformed urinary NT-proBNP concentration was significantly associated with a 20% (95% CI: 7–35%) greater risk of CVD, whereas there was no clear evidence of heterogeneity in the association of every 1-SD increment in log-transformed urinary NT-proBNP concentration with the risk of CVD between the subgroups of traditional cardiovascular risk factors (all *P* for heterogeneity > 0.1), except for the use of antihypertensive agents and eGFR levels (Table 3). The association between urinary NT-proBNP concentration and the risk of CVD tended to be weaker in the subjects using antihypertensive agents than in those not using them, and in subjects with eGFR of < 60 mL/min/1.73 m^2^ than in those with eGFR of ≥ 60 mL/min/1.73 m^2^ (both *P* for heterogeneity ≤ 0.08). Heterogeneities were detected in the association with CHD events between the two antihypertensive-agent subgroups (*P* for heterogeneity = 0.03) and in the association with stroke events between the two eGFR-level subgroups (*P* for heterogeneity = 0.10) (Supplementary Table 2).

**Table 3:** Multivariable-adjusted hazard ratios of the log-transformed urinary NT-proBNP for the development of cardiovascular disease, stratified by presence or absence of traditional cardiovascular risk factors

Subgroups	Persons at risk	No. of events	Hazard ratio (95% CI) per 1-SD increment in log-transformed (log_10_) urinary NT-proBNP^a^	*P* for heterogeneity
**Overall**	3055	169	1.20 (1.07–1.35)	
**Age**				
40–64 years	1691	32	1.21 (0.75–1.96)	0.44
≥ 65 years	1364	137	1.25 (1.11–1.41)	
**Sex**				
Men	1286	92	1.21 (1.03–1.41)	0.48
Women	1769	77	1.30 (1.08–1.57)	
**Hypertension**				
No	1612	47	1.39 (1.06–1.81)	0.11
Yes	1443	122	1.17 (1.02–1.33)	
**Use of antihypertensive agents**				
No	2161	89	1.30 (1.09–1.56)	0.08
Yes	894	80	1.16 (0.98–1.37)	
**Diabetes**				
No	2597	122	1.26 (1.11–1.43)	0.24
Yes	458	47	0.96 (0.71–1.29)	
**Hypercholesterolemia**				
No	1610	79	1.20 (1.03–1.40)	0.54
Yes	1445	90	1.13 (0.95–1.35)	
**Use of lipid-lowering drugs**				
No	2630	130	1.19 (1.03–1.37)	0.65
Yes	425	39	1.34 (1.07–1.67)	
**Body mass index (kg/m**^**2**^**)**				
< 25	2268	117	1.25 (1.09–1.42)	0.38
≥ 25	787	52	1.18 (0.93–1.51)	
**eGFR level (mL/min/1.73 m**^**2**^**)**				
≥ 60	2742	130	1.30 (1.10–1.53)	0.07
< 60	313	39	1.18 (1.00–1.39)	

The SD of log-transformed urinary NT-proBNP levels (pg/mL) was 0.215

*Abbreviations*: *SD* Standard deviation, *NT-proBNP* N-terminal pro–B-type natriuretic peptide, *CI* Confidence interval, *eGFR* Estimated glomerular filtration rate

^a^Adjusted for age, sex, systolic blood pressure, antihypertensive agents, diabetes mellitus, serum total and high-density lipoprotein cholesterol levels, lipid-lowering agents, body mass index, electrocardiogram abnormality, estimated glomerular filtration rate, smoking habits, alcohol intake, and regular exercise. The variable relevant to the subgroup was excluded from each model. Five participants with missing data for covariates were excluded from this analysis (*n* = 3055)

## Discussion

The present study clearly demonstrated a strong quadratic correlation between the serum and urinary concentrations of NT-proBNP in a general Japanese population. We further confirmed a significant association of elevated urinary NT-proBNP concentrations with greater risk of total CVD including stroke in a general population after adjusting for conventional cardiovascular risk factors. These findings suggest that the measurement of urinary NT-proBNP concentrations could be a surrogate for serum NT-proBNP levels and highlight the potential value of the measurement of urinary NT-proBNP concentration for the risk assessment of CVD in a general Japanese population.

Several hospital-based studies have shown a strong positive correlation of urinary NT-proBNP with serum/plasma NT-proBNP in patients with heart failure or hypertension [12–21]. The present study confirmed the strong positive correlation between urinary and serum NT-proBNP levels in a general population, who were likely to have lower serum NT-proBNP concentrations than patients with heart failure or hypertension. In addition, a similar positive correlation between urinary and serum values was observed in the subgroups with and without kidney dysfunction. Serum and urinary NT-proBNP levels may be affected by renal dysfunction because serum NT-proBNP is known to be filtered in the glomeruli and excreted in urine. Several previous studies reported that serum NT-proBNP concentrations increased with lower eGFR levels and discussed that this finding might be due to decreased clearance [28–30]. On the other hand, a clinical study has shown that urinary NT-proBNP concentration is affected by renal plasma flow but not by eGFR [11]. Furthermore, the present study found a significant positive association between urinary NT-proBNP concentration and cardiovascular risk in both subgroups of eGFR levels, although the association was slightly attenuated in subjects with lower eGFR. The findings from the present study suggest that urinary NT-proBNP concentration could be a surrogate index of serum NT-proBNP levels, even in subjects with mild–moderate reduction of eGFR.

Self-monitoring and management of the cardiovascular risk factors have become increasingly important for improving the clinical outcomes of subjects at higher risk of cardiovascular events in the community and for patients with heart failure [22, 31]. In the present study, higher urinary NT-proBNP levels were associated with higher risk of cardiovascular events in a general population. A hospital-based prospective study previously showed that elevated urinary NT-proBNP levels were associated with a higher risk of cardiac events (mortality and admission) in patients with heart failure [14]. Another study reported that higher urinary NT-proBNP levels were associated with subsequent emergency department visits in chronic heart failure patients [21]. In a community-screening study in the UK, urinary NT-proBNP concentrations had good discrimination ability for detecting left ventricular systolic dysfunction [32]. Because urine sampling is noninvasive and does not require specially trained personnel, these findings raise the possibility that the repeated measurement of urinary NT-proBNP concentration could help in regular self-monitoring and management for the early identification and treatment of subclinical cardiac damage in communities, especially for people with difficulty in access to the clinics or hospitals (i.e., elderly or disabled individuals).

In the present study, higher urinary NT-proBNP concentrations were associated significantly with increased risk of stroke, while there was no evidence of a significant association for CHD. The same was true in the analysis of serum NT-proBNP concentrations (Supplementary Table 3). In our previous study in an earlier cohort of community residents in Hisayama, the risks of both CHD and stroke increased significantly with elevating serum NT-proBNP concentrations [5]. The exact reason for the discrepancy in the influence of serum NT-proBNP concentrations for CHD event was unclear, but it might be partially explained by the increasingly widespread use of antihypertensive medications. The association of elevated urinary NT-proBNP levels with the risk of CHD was observed in participants without antihypertensive treatment but was less evident in participants with antihypertensive agents. Consequently, the association of urinary NT-proBNP levels with CHD risk may have been weakened by the spread of antihypertensive drugs.

Several limitations should also be noted. First, urinary NT-proBNP concentrations in the present study were measured using spot urine samples collected mainly at the center for the health check-up in the morning. The time of urine collection and the statuses of water intake and medication were not standardized among the participants. The differences in these factors might misclassify the urinary NT-proBNP levels, likely underestimating the association between urinary NT-proBNP levels and the cardiovascular risk. In addition, the measurement of urinary NT-proBNP concentrations was based on a single measurement using spot urine sampling at baseline, and there were no data on the urinary NT-proBNP concentrations during the follow-up period. Furthermore, the Elecsys proBNP Immunoassay (Roche Diagnostics) was originally designed for use with serum and plasma samples, and its validity for use with urinary samples has not been fully established. These limitations might have resulted in misclassification of the urinary NT-proBNP levels. Such misclassification could have weakened the association, biasing the results toward the null hypothesis. Second, we measured urinary NT-proBNP levels using frozen samples that were stored for more than 10 years. A previous study showed a strong correlation between fresh and frozen urine NT-proBNP levels in patients with chronic heart disease [18]. Another study reported that plasma concentration of NT-proBNP was stable after frozen storage for approximately 2 years [33]. However, the stability of urinary NT-proBNP levels for more than 10 years has not been established. Long-term storage might degrade NT-proBNP and possibly decrease NT-proBNP concentration in samples. However, such degradations might occur randomly among the samples, possibly resulting that the misclassification in the urinary NT-proBNP levels were likely to weaken the association between urinary NT-proBNP levels and cardiovascular risk. Therefore, the influence of this issue on the findings may be modest. Third, we could not address the correlation between urinary and serum NT-proBNP concentration in subjects with severe reduction of eGFR due to the small sample size (*n* = 14 [0.46%] with eGFR < 30 mL/min/1.73 m^2^). Moreover, the correlation between the serum and urinary concentrations of NT-proBNP was relatively high (*R*^2^ = 0.72 in the quadratic regression, Supplementary Table 1), but the observed urinary concentrations of NT-proBNP were deviated from the estimated values using the regression curve in approximately 13% of the subjects (≥ + 10% or ≤ − 10% in the proportional difference; *n* = 351). These subjects were likely to be older and more likely to have hypertension, electrocardiogram abnormality, reduced eGFR, and higher serum NT-proBNP levels (data not shown). Urinary NT-proBNP levels in the elderly or in people with hypertension or cardiac or kidney dysfunction might be less accurate as a marker for the cardiovascular risk. Further large-scale studies are needed to elucidate this issue. Finally, the generalizability of our findings is limited because this study was conducted in a small town in Japan. These issues should be addressed in other large-scale studies of general populations with various genetic and lifestyle backgrounds.

## Conclusions

Our findings suggest that urinary NT-proBNP concentrations were well-correlated with serum concentrations and were positively associated with cardiovascular risk. The urinary NT-proBNP concentrations have the potential to be a biomarker for detecting people with higher cardiovascular risk.

## Supplementary Information

**Additional file 1: Supplementary Table 1.** The regression equation and the coefficient of determination (*R*^2^) between the serum and urinary NT-proBNP concentrations according to the various regression models. **Supplementary Table 2.** Multivariable-adjusted hazard ratios for the association between log-transformed urinary NT-proBNP concentrations and the risk of developing cardiovascular subtypes according to the use or nonuse of antihypertensive agents and high or low eGFR levels. **Supplementary Table 3.** Age- and sex-adjusted and multivariable-adjusted hazard ratios for the association between serum NT-proBNP levels and the risk of developing cardiovascular disease and its subtypes.

## Data Availability

The datasets generated and/or analyzed during the current study are not publicly available due to restrictions included in the informed consent of research participants.

## Abbreviations

NT-proBNP: N-terminal pro–B-type natriuretic peptide
CVD: Cardiovascular disease
CHD: Coronary heart disease
eGFR: Estimated glomerular filtration rate
BMI: Body mass index
