# Supplementary material for: Urinary N-terminal pro–B-type natriuretic peptide as a biomarker for cardiovascular events in a general Japanese population: the Hisayama Study
Source: Environ Health Prev Med. 2021 Apr 12;26:47. doi: 10.1186/s12199-021-00970-0 (PMC8042718; doi:10.1186/s12199-021-00970-0)
Supplement: Supplementary file 1 — Additional file 1: Supplementary Table 1. The regression equation and the coefficient of determination (R2) between the serum and urinary NT-proBNP concentrations according to the various regression models. Supplementary Table 2. Multivariable-adjusted hazard ratios for the association between log-transformed urinary NT-proBNP concentrations and the risk of developing cardiovascular subtypes according to the use or nonuse of antihypertensive agents and high or low eGFR levels. Supplementary Table 3. Age- and sex-adjusted and multivariable-adjusted hazard ratios for the association between serum NT-proBNP levels and the risk of developing cardiovascular disease and its subtypes. [file 12199_2021_970_MOESM1_ESM.docx]

**Supplementary Table 1.** The regression equation and the coefficient of determination (R^2^) between the serum and urinary NT-proBNP concentrations according to the various regression models.

|  | x | y | Regression equation | R^2^ |
| --- | --- | --- | --- | --- |
| Model 1 | Serum NT-proBNP, pg/mL | Urinary NT-proBNP, pg/mL | y = 0.213x + 4.349 | 0.513 |
| Model 2 | log_10_ (serum NT-proBNP), pg/mL | log_10_ (urinary NT-proBNP), pg/mL | y = 0.363x + 0.716 | 0.562 |
| Model 3 | log_10_ (serum NT-proBNP), pg/mL | log_10_ (urinary NT-proBNP), pg/mL | y = 0.250x^2^ - 0.602x + 1.593 | 0.722 |

Abbreviations: NT-proBNP, N-terminal pro-brain natriuretic peptide.

Model 1: a linear regression between the serum and urinary NT-proBNP levels.

Model 2: a linear regression between the log-transformed serum and urinary NT-proBNP levels.

Model 3: a quadratic regression between the log-transformed serum and urinary NT-proBNP levels.

**Supplementary Table 2.** Multivariable-adjusted hazard ratios for the association between log-transformed urinary NT-proBNP concentrations and the risk of developing cardiovascular subtypes according to the use or nonuse of antihypertensive agents and high or low eGFR levels

| Subgroups | | Persons at risk | No. of events | Hazard ratio (95% CI) per 1-SD increment in log-transformed (log_10_) urinary NT-proBNP^a^ | *P* for heterogeneity |
| --- | --- | --- | --- | --- | --- |
| ***Coronary heart disease*** | | | | | |
| **Overall** | | 3,055 | 77 | 1.10 (0.89-1.35) |  |
| **Use of antihypertensive agents** | | | | | |
|  | No | 2,161 | 39 | 1.39 (1.05-1.85) | 0.03 |
|  | Yes | 894 | 38 | 0.91 (0.64-1.27) |  |
| **eGFR level (mL/min/1.73 m^2^)** | | | | | |
|  | ≥60 | 2,742 | 60 | 1.16 (0.85-1.57) | 0.47 |
|  | <60 | 313 | 17 | 1.09 (0.79-1.50) |  |
| ***Stroke*** | | | | | |
| **Overall** | | 3,055 | 94 | 1.24 (1.08-1.43) |  |
| **Use of antihypertensive agents** | | | | | |
|  | No | 2,161 | 51 | 1.24 (0.99-1.54) | 0.51 |
|  | Yes | 894 | 43 | 1.28 (1.04-1.56) |  |
| **eGFR level (mL/min/1.73 m^2^)** | | | | | |
|  | ≥60 | 2,742 | 71 | 1.34 (1.09-1.64) | 0.10 |
|  | <60 | 313 | 23 | 1.19 (0.99-1.44) |  |

Abbreviations: SD, standard deviation; NT-proBNP, N-terminal pro-brain natriuretic peptide; CI, confidence interval; eGFR, estimated glomerular ﬁltration rate.

The SD of log-transformed urinary NT-proBNP levels (pg/mL) was 0.215.

^a^Adjusted for age, sex, systolic blood pressure, antihypertensive agents, diabetes mellitus, serum total and high-density lipoprotein cholesterol levels, lipid-lowering agents, body mass index, electrocardiogram abnormality, estimated glomerular ﬁltration rate, smoking habits, alcohol intake and regular exercise. The variable relevant to the subgroup was excluded from each model. Five participants with missing data for covariates were excluded from this analysis.

**Supplementary Table 3.** Age- and sex-adjusted and multivariable-adjusted hazard ratios for the association between serum NT-proBNP levels and the risk of developing cardiovascular disease and its subtypes

| Serum  NT-proBNP  (pg/mL) | Persons at risk | No. of events | Age- and sex-adjusted | |  | Multivariable-adjusted^a^ | |
| --- | --- | --- | --- | --- | --- | --- | --- |
|  |  |  | Hazard ratio  (95% CI) | *P* value |  | Hazard ratio  (95% CI) | *P* value |
| **Cardiovascular disease** | | | | | | | |
| <55 | 1,494 | 53 | 1.00 (reference) |  |  | 1.00 (reference) |  |
| 55-124 | 956 | 41 | 0.75 (0.48-1.16) | 0.19 |  | 0.80 (0.52-1.24) | 0.32 |
| 125-299 | 419 | 40 | 1.23 (0.76-1.98) | 0.40 |  | 1.24 (0.76-2.03) | 0.38 |
| ≥300 | 191 | 36 | 2.23 (1.34-3.71) | 0.002 |  | 1.83 (1.05-3.20) | 0.03 |
| *P* for trend |  |  |  | <0.001 |  |  | 0.02 |
| **Coronary heart disease** | | | | | | | |
| <55 | 1,494 | 31 | 1.00 (reference) |  |  | 1.00 (reference) |  |
| 55-124 | 956 | 20 | 0.73 (0.40-1.34) | 0.31 |  | 0.81 (0.44-1.49) | 0.49 |
| 125-299 | 419 | 16 | 1.03 (0.51-2.08) | 0.92 |  | 1.08 (0.52-2.23) | 0.84 |
| ≥300 | 191 | 11 | 1.45 (0.65-3.21) | 0.36 |  | 1.21 (0.49-2.97) | 0.67 |
| *P* for trend |  |  |  | 0.38 |  |  | 0.64 |
| **Stroke** | | | | | | | |
| <55 | 1,494 | 23 | 1.00 (reference) |  |  | 1.00 (reference) |  |
| 55-124 | 956 | 21 | 0.80 (0.42-1.49) | 0.48 |  | 0.81 (0.43-1.53) | 0.52 |
| 125-299 | 419 | 25 | 1.51 (0.79-2.91) | 0.21 |  | 1.48 (0.76-2.86) | 0.25 |
| ≥300 | 191 | 25 | 2.98 (1.49-5.94) | 0.002 |  | 2.31 (1.10-4.85) | 0.03 |
| *P* for trend |  |  |  | <0.001 |  |  | 0.01 |

Abbreviations: NT-proBNP, N-terminal pro-brain natriuretic peptide; CI, confidence interval.

^a^Adjusted for age, sex, systolic blood pressure, antihypertensive agents, diabetes mellitus, serum total and high-density lipoprotein cholesterol levels, lipid-lowering agents, body mass index, electrocardiogram abnormality, estimated glomerular ﬁltration rate, smoking habits, alcohol intake and regular exercise. Five participants with missing data for covariates were excluded from the multivariable-adjusted analysis (n=3,055).
